# Flipped binding modes for the same agonist in closely related neuropeptide-gated ion channels

**DOI:** 10.1016/j.bpj.2025.01.004

**Published:** 2025-01-11

**Authors:** Emily J.S. Claereboudt, Mowgli Dandamudi, Léa Longueville, Hassan Y. Harb, Timothy Lynagh

**Affiliations:** 1 Michael Sars Centre, University of Bergen, Bergen, Norway; 2 Concept Life Sciences Limited, Chapel-en-le-Frith, UK

## Abstract

Neuropeptides are inter-cellular signaling molecules occurring throughout animals. Most neuropeptides bind and activate G-protein-coupled receptors, but some also activate ionotropic receptors (or “ligand-gated ion channels”). This is exemplified by the tetra-peptide H-Phe-Met-Arg-Phe-NH_2_ (FMRFamide (FMRFa)), which activates mollusk and annelid FMRFa-gated sodium channels (FaNaCs) from the trimeric degenerin/epithelial sodium channel superfamily. Here, we explored the structure-activity relationships determining FMRFa potency at mollusk and annelid FaNaCs in the light of emerging structural data, using synthetic neuropeptide analogs, heterologous expression, and two-electrode voltage clamp. Substitutions of the FMRFa N-terminal phenylalanine residue (F1) and methionine residue (M2) decreased or abolished FMRFa potency at mollusk *Aplysia kurodai* FaNaC but had little effect at annelid *Malacoceros fuliginosus* FaNaC1. Conversely, F4 substitutions had little effect on FMRFa potency at *A. kurodai* FaNaC but either abolished, strongly decreased, or slightly increased potency at *M. fuliginosus* FaNaC1. Accordingly, recently published high-resolution FaNaC structures show that F1 and F4 residues orient deep into the neuropeptide-binding pockets of *A. kurodai* FaNaC and *M. fuliginosus* FaNaC1, respectively. We also use noncanonical amino acid substitutions in *A. kurodai* FaNaC to describe the physico-chemical determinants of FMRFa F1 binding to *A. kurodai* FaNaC aromatic side chains. Our results show that the “deeper” of the two FMRFa phenylalanine residues in the binding pocket is crucial for FMRFa potency despite the peptide orienting very differently into the homologous binding sites of two closely related receptors.

## Significance

In annelid worms and mollusks, the neuropeptide FMRFamide (FMRFa) activates ligand-gated sodium channels (FaNaCs) in the cell membrane, leading to sodium influx and neuronal excitation. We examined the structure-activity relationships determining FMRFa potency at mollusk and annelid FaNaCs in the light of recently emerged structural data. We find that one end of the FMRFa ligand determines potency at one FaNaC and the opposite end determines potency at the other FaNaC, illustrating a curious case of the same ligand binding in flipped orientations in two closely related receptors.

## Introduction

Neuropeptides, derived from longer propeptides or precursor proteins, are relatively small proteinaceous molecules that are packaged and released by one cell to bind and activate protein targets on another cell (1). They are widely distributed in both invertebrate and vertebrate animals and act mainly as neurotransmitters and neuromodulators (1). The tetra-peptide H-Phe-Met-Arg-Phe-NH_2_ (FMRFamide (FMRFa)) is a cardioexcitatory peptide in mollusks (2) that is notable because of the wide use of FMRFa immunoreactivity for imaging invertebrate nervous system morphology (3). Although propeptides specifically yielding FMRFa are found in only two phyla, Mollusca and Annelida, FMRFa-like neuropeptides (FLPs) are found in most major phyla, such as FLRFa in mollusks and DPKQDFMRFa in insects, and similar peptides in vertebrates including neuropeptide FF, ending with RFamide (3).

Most neuropeptide receptors are G-protein-coupled receptors (GPCRs), but other targets include certain tyrosine kinase receptors in numerous animals and peptide-gated ion channels in invertebrates (4). For FMRFa or FLPs, two major families of receptors have been identified and functionally characterized. One is a GPCR, homologs of which have been described (e.g., for insects including the fruit fly *Drosophila melanogaster* (5) and for the annelid worm *Platynereis dumerilii* (6)). The other is the FMRFa-gated Na^+^ channel (FaNaC) family, proteins via which FMRFa directly activates excitatory Na^+^ flux(7,8), and FaNaCs from both mollusks and annelids have been described (8,9). FaNaCs are members of the degenerin/epithelial sodium channel (DEG/ENaC) superfamily of amiloride-sensitive trimeric Na^+^ channels. In contrast to FaNaCs, the DEG/ENaC channels found in mammals are gated by increased proton concentrations (acid-sensing ion channels [ASICs]), bile acid (bile acid-sensitive ion channels), or are constitutively active (ENaCs) (10). However, FMRFa and similar RFamides have been shown to bind to ASICs and modulate their activity (11,12,13).

Functional and phylogenetic analysis of the FaNaC family identified two closely related but distinct clades of FaNaCs (9). The first (clade 1) includes mollusk, annelid, and brachiopod genes, several of which encode channels gated by FMRFa and similar peptides from FMRFa propeptides. The second clade (clade 2) includes annelid genes that encode channels gated by FMRFa and/or several peptides from FMRFa and other propeptides, such as FVRIa, RYa, and Wa (or myoinhibitory peptide). Two recent cryoelectron microscopy (cryo-EM) studies solved structures of FMRFa-bound FaNaCs, one from clade 1 (14) and one from clade 2 (15), and these structures raise two intriguing notions. Firstly, despite the close phylogenetic relation of the two receptors and the shared location of the FMRFa-binding site at the extracellular “corner” of the protein, the tetra-peptide appears to orient very differently into the binding sites in the two receptors. Secondly, the site in mammalian ASICs via which FMRFa and other peptides are believed to modulate activity is located far from the external corner, closer to the channel pore (11,12,13), again suggesting substantial diversity in binding sites for FMRFa.

Here, we sought experimental evidence for the apparent diversity in FMRFa binding to closely related receptors and tried to establish the determinants of FMRFa potency in diverse FaNaCs. We therefore tested the activity of numerous synthetic FMRFa analogs at *Aplysia kurodai* FaNaC (clade 1) and *Malacoceros fuliginosus* FaNaC1 (clade 2). This structure-activity analysis, together with mutagenesis of crucial amino acid residues in the channels, provides convincing evidence for flipped binding modes of the same peptide in two closely related DEG/ENaC channels.

## Materials and methods

### Plasmids and molecular biology

*A. kurodai* FaNaC (NCBI GenBank: AB206707.1) and *M. fuliginosus* FaNaC1 (NCBI GenBank: ON156825.1) cDNAs in tailored pSP64 plasmid vectors including *Xenopus laevis* UTRs and poly(A) sequences have been described at length previously (9,16). *A. kurodai* FaNaC F188Y, F188tag (amber stop codon), F453Y, and F453tag mutants were generated by site-directed mutagenesis as detailed in (17) with custom primers (Merck) and PCR with Phusion High-Fidelity DNA polymerase (Thermo Fisher). All coding sequences were confirmed by Sanger sequencing (Genewiz). Plasmid DNA was linearized by digesting with EcoRI (Thermo Fisher) and purified with the DNA Clean & Concentrator Kit (Zymo). mRNA was then transcribed using the mMESSAGE mMACHINE SP6 Transcription Kit (Thermo Fisher) and purified with the RNeasy Mini Kit (Qiagen).

### Peptide and noncanonical amino acid synthesis

All peptides were custom synthesized by Genscript confirmed with electrospray ionization mass spectrometry and ≥95% purity confirmed by reversed-phase high-performance liquid chromatography (HPLC), and trifluoric acid replaced with acetic acid. Phenylalanine (Phe) and noncanonical derivatives homophenylalanine (hPhe) and cyclohexylalanine (Cha) were synthesized as aminoacylated RNA dinucleotide (OpdCpA) ditetrabutylammonium salts with the amino acid amine protected with a 4,5-dimethoxy-2-nitrobenzyloxycarbonyl (Nvoc) protecting group. Ditetrabutylammonium Nvoc-Phe-OpdCpA, ditetrabutylammonium Nvoc-hPhe-OpdCpA, and ditetrabutylammonium Nvoc-Cha-OpdCpA were synthesized and verified as described in the Supplemental Materials and Methods (Figs. S1–S9). pdCpA was synthesized according to (18).

### Heterologous expression and noncanonical amino acid incorporation

*X. laevis* frog oocytes were shipped from Ecocyte Bioscience, Germany, and stored at 18°C in 50% Leibovitz’s L-15 medium (Gibco) supplemented with additional 0.25 mg/mL gentamicin, 1 mM L-glutamine, and 15 mM HEPES, pH 7.6.

Noncanonical amino acids were incorporated into positions F188 and F453 of *A. kurodai* FaNaC via a nonsense suppression approach using an amber stop codon (TAG/UAG) and modified *Tetrahymena thermophila* Gln73 tRNA (THG73) (19,20). In short, THG73 was prepared by annealing forward and reverse DNA oligos (Merck) encoding THG73 with a T7 promotor. The resulting double-stranded DNA (dsDNA) was purified and concentrated by ethanol precipitation. THG73 RNA was transcribed with the T7-Scribe Transcription Kit (Cellscript) and purified in Chroma Spin DEPC-H20 columns (Clontech). tRNA was folded by heating to 95°C for 3 min and cooling to 50°C and then immediately ligated to the aminoacylated dinucleotides, with T4 RNA ligase (New England Biolabs). Aminoacylated tRNA was purified with phenol-chloroform extraction and ethanol precipitation, dried, and the pellet stored at −80°C until use.

For wild-type (WT) and conventional mutant channels, oocytes were injected with 40 nL of 120 ng/*μ*L FaNaC mRNA via glass micropipettes backfilled with mineral oil, using a Nanoliter2010 injector (World Precision Instruments). For the expression of noncanonical mutant channels, aminoacylated tRNA was first resuspended in 2 *μ*L of water and deprotected by 5 min in UV light via a fan-cooled coil of 365-nm realUV LED strip lights (Waveform Lighting). Deprotected aminoacylated tRNA was then mixed 2:1 with 700 ng/*μ*L UAG mutant FaNaC mRNA, and 40 nL of this mix was injected into oocytes.

### Electrophysiology and data analysis

Two-electrode voltage clamp experiments were performed 24–36 h after injection. The oocyte was placed in an RC-3Z bath (Warner Instruments) and perfused continuously with ND96 solution (NaCl 96 mM, KCl 2 mM, CaCl_2_ 1.8 mM, MgCl_2_ 1 mM, HEPES 5 mM, pH 7.5 with NaOH). In experiments without Ca^2+^, CaCl_2_ was replaced with BaCl_2_, and oocytes were injected with 40 nL of water or 25 mM EGTA 10–45 min before recording. In experiments probing increased Ca^2+^ concentrations, extracellular solutions contained NaCl 140 mM, HEPES 10 mM, CaCl_2_ 1 mM, pH 7.5 with NaOH; or NMDG-Cl 126.5 mM, HEPES 10 mM, CaCl_2_ 10 mM, pH 7.5 with NaOH (leaving a small amount of Na^+^ in the final solution), based on previous work (21). ND96 (or the latter solutions) alone or containing peptides was rapidly exchanged via a VCS-8 pinch-valve control perfusion system (Warner Instruments). Peptides at concentrations ranging from 0.1 nM to 100 *μ*M, as indicated in figures, were applied for 5–10 s and oocytes were washed for 1–5 min between subsequent applications depending on peptide and channel. Oocytes were clamped at −80 mV for all experiments with an Oocyte Clamp OC-725D amplifier (Warner Instruments) and Axon Digidata 1550B digitizer (HEKA Elektronik). Data were recorded at 1000 Hz and filtered at 100–200 Hz. 50 Hz noise was eliminated with a Hum Bug (Digitimer). In measuring reversal potentials in normal and high Ca^2+^ solutions, 150 ms voltage ramps from −80 mV to 80 mV were applied continuously, before and during FMRFa application. The resulting “current ramp” in the absence of FMRFa was subtracted from that during peak FMRFa-gated current to plot the FMRFa-induced current-voltage relationship.

Current amplitude was measured in Clampfit 11.1 (Molecular Devices), and subsequent data analyses were performed in Prism v9 (GraphPad Software). For peptide concentration-response data, peak current amplitude was normalized to maximum, plotted against peptide concentration, and fitted with four-parameter nonlinear regression (Prism v9) for each oocyte. These were averaged to give the reported means ± SE in the main text. For display in figures, a single fit to the average normalized responses (±SE) is shown. Deactivation of *M. fuliginosus* FaNaC1 after LMRFa application was especially slow. Therefore, in most *M. fuliginosus*/LMRFa experiments only, just two LMRFa applications were used: one test concentration and one 10 *μ*M for normalization. This yielded *n* = 3 oocytes for responses to different concentrations, against which a single curve was fitted and half-maximal effective concentration (EC_50_) was calculated. Multiple comparisons were made with one-way ANOVA with Dunnett’s comparison to a control value (e.g., comparing with FMRFa).

### Immunolabeling

Oocytes injected with different constructs or water as negative control were fixed with 4% paraformaldehyde diluted in phosphate-buffered saline (PBS) overnight at 4°C. Oocytes were then embedded in 3% low-gelling-point agarose at 4°C for 2 h, sliced with a vibratome in 100 *μ*m sections, and blocked for 3 h in a PBS-based solution containing 0.2% bovine serum albumin (BSA) and 0.1% Tween 20. A c-Myc tag (EQKLISEEDL) in the C terminus of FaNaC constructs was detected by incubating the slices overnight at 4°C with a mouse anti-c-Myc monoclonal IgG1 antibody (MA121316, Fisher Scientific) diluted 1:500 in blocking buffer (1% BSA and 0.1% Tween 20 in PBS). Slices were then incubated for 1 h at room temperature with goat anti-mouse polyclonal immunoglobulin (Ig)G (H + L) Alexa Fluor 568 conjugate (A11004, Thermo Fisher) diluted 1:1000 in blocking buffer. Slices were mounted on glass slides using VECTASHIELD Antifade Mounting Medium (H-1000-10, Vector Laboratories). Oocytes or slices were washed several times with PBS between the different steps. Images were acquired using a Zeiss Axio Scope A1 microscope with a 40× objective.

## Results

### Experimental characterization of *A. kurodai* FaNaC and *M. fuliginosus* FaNaC1

**Figure 1:**
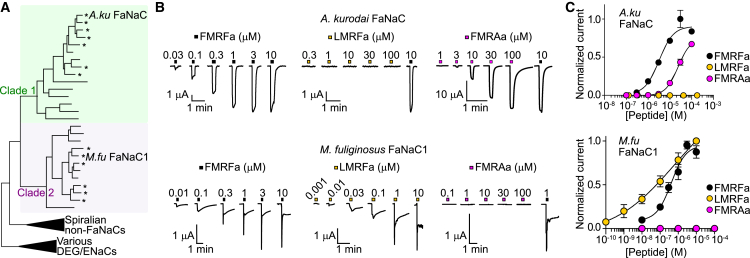
Measuring peptide potency at a clade 1 FaNaC and a clade 2 FaNaC. (*A*) FaNaC branch from previously published DEG/ENaC phylogeny (9) showing clade 1 in green and clade 2 in purple. Asterisks indicate previously verified FMRFa-gated or similar peptide-gated channels. *A. kurodai* (*A.ku*) and *M. fuliginosus* (*M.fu*) FaNaC amino acid sequences shown in Fig. S11. (*B*) Example two-electrode voltage clamp recordings of inward currents in response to indicated peptides in oocytes expressing *A.ku* FaNaC (*top*) or *M. fu* FaNaC1 (*bottom*). (*C*) Mean ± SE normalized (to maximum) current amplitudes (*n* = 3–5) in response to increasing concentrations of indicated peptides. In most *M. fuliginosus* FaNaC1/LMRFa experiments only, just two LMRFa applications were used at each oocyte: one test concentration and 10 *μ*M for normalization; a single curve was fitted to the six data points.

We aimed to assess the structure-activity of FMRFa at representative channels from the two distinct clades of the FaNaC family. For this we chose *A. kurodai* FaNaC, a mollusk channel from clade 1, and *M. fuliginosus* FaNaC1, an annelid channel from clade 2 (Figs. 1 *A* and S4). We expressed these channels in *X. laevis* oocytes and measured current responses to FMRFa and other peptides via two-electrode voltage clamp (Fig. 1 *B*). FMRFa activated large currents in both channels, with an EC_50_ of 5.48 ± 0.05 *μ*M at *A. kurodai* FaNaC and 0.35 ± 0.06 *μ*M at *M. fuliginosus* FaNaC1 (Fig. 1 *B* and *C*), similar to previous reports for these channels (9,16).

Although both FaNaCs are potently activated by FMRFa, current kinetics were significantly different. Although *A. kurodai* FaNaC showed rapid activation, continued current in the presence of agonist, and then rapid deactivation after removal of the peptide, *M. fuliginosus* FaNaC1 desensitized rapidly in the presence of agonist and deactivation was slow, requiring >4 min between peptide applications for concentration-response experiments, as reported in previous work (15). *M. fuliginosus* FaNaC1 currents showed rapid desensitization even in the absence of Ca^2+^, and high extracellular Ca^2+^ concentrations in the absence of Na^+^ did not lead to large currents (Fig. S10). Therefore, we think FaNaC1 is mostly Na^+^ permeable (15), and rapid desensitization is intrinsic to FaNaC1 dynamics and not a result of potential Ca^2+^ permeation activating endogenous Ca^2+^-activated Cl^−^ channels in oocytes (22).

### Different residues of the tetra-peptide determine potency at *A. kurodai* FaNaC and *M. fuliginosus* FaNaC1

Previous experiments on various mollusk FaNaCs show that clade 1 FaNaCs are also activated by peptides closely related to FMRFa, such as FLRFa and FMKFa (7,9). In contrast, previous experiments on clade 2 channels from various annelids suggest that some clade 2 FaNaCs are additionally or instead activated by peptides from different propeptides, including ASSFVRIa, LFRYa, and even larger “Wamides” or “myoinhibitory peptides” (9,23). We sought to identify the determinants of peptide potency at clade 1 and clade 2 FaNaCs by testing 15 synthetic peptides differing from FMRFa at the F1 residue or N terminus, at the M2 residue, at the R3 residue, or at the F4 residue or C terminus.

**Figure 2:**
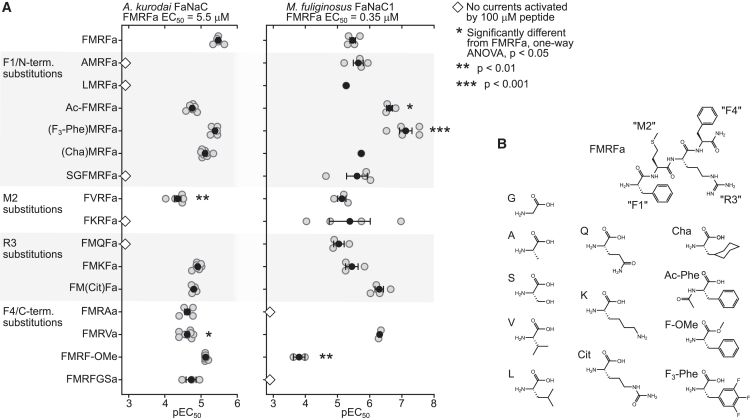
Potency of synthetic FMRFa analogs at *A. kurodai* FaNaC and *M. fuliginosus* FaNaC1. (*A*) Increasing peptide concentrations were applied to oocytes expressing indicated FaNaCs, and EC_50_ values were calculated for each oocyte and converted to pEC_50_ (−logEC_50_). Gray symbols, pEC_50_ values at different oocytes: *n* = 2 for FMRF-OMe at *M. fuliginosus* FaNaC1; *n* = 3–6 for all others. Black symbols, mean ± SE. Ac, N-terminal acetyl; F_3_-Phe, 3,4,5-trifluorophenylalanine; Cha, cyclohexylalanine; Cit, citrulline; OMe, C-terminal O-methylester. In most *M. fuliginosus* FaNaC1/LMRFa experiments only, just two LMRFa applications were used: one test concentration, and 10 *μ*M for normalization, and a single curve/EC_50_ was calculated. (*B*) 2D structures (nonionized form) of FMRFa and amino acids used in FMRFa analogs.

Alterations to the F1 residue or the peptide N terminus had substantially different effects on potency at *A. kurodai* FaNaC compared to *M. fuliginosus* FaNaC1. For example, LMRFa and AMRFa, in which the large aryl F1 side chain is replaced with smaller hydrophobic side chains, activated no current at *A. kurodai* FaNaC but activated *M. fuliginosus* FaNaC1 similarly to FMRFa (Figs. 1 *B* and *C* and 2 *A*). Replacing F1 with tri-fluorophenylalanine (in the peptide (F_3_-Phe)MRFa) or cyclohexylalanine (in (Cha)MRFa) did not significantly decrease potency at *A. kurodai* FaNaC (Fig. 2 *A*). F_3_-Phe and Cha side chains have decreased electron density in the middle of the aromatic ring and nonplanar geometry, respectively, compared to phenylalanine, but are otherwise similarly sized and hydrophobic (Fig. 2 *B*). Thus, F1 size and hydrophobicity, but not electron delocalization or planar geometry, are important for FMRFa potency at *A. kurodai* FaNaC. At *M. fuliginosus* FaNaC1, (Cha)MRFa acted similarly to FMRFa, whereas (F_3_-Phe)MRFa showed significantly greater potency than FMRFa (Fig. 2), with an EC_50_ of 11 ± 5 nM (*n* = 5), compared to 350 ± 67 nM for FMRFa. Finally, N-terminal acetylation (Ac-FMRFa in Fig. 2) or serine-glycine insertion (SGFMRFa in Fig. 2) had little effect or abolished activity at *A. kurodai* FaNaC but led to increased potency or no effect on *M. fuliginosus* FaNaC1 (Fig. 2). Thus, F1 and to some extent a short N terminus (without amino acid residues before F1) are required for FMRFa activity at *A. kurodai* FaNaC, but at *M. fuliginosus* FaNaC1 neither of these are necessary for FMRFa activity and their substitution can in fact enhance potency.

Methionine side chains are similarly hydrophobic to valine (V) but closer in shape to polar lysine (K; Fig. 2 *B*). FVRFa was significantly less potent than FMRFa, and FKRFa had no activity, at *A. kurodai* FaNaC (Fig. 2 *A*). In contrast, replacing M2 with a shorter hydrophobic side chain (FVRFa) or a similarly sized but polar side chain (FKRFa) had no significant effect on potency at *M. fuliginosus* FaNaC1 (Fig. 2 *A*). The R3 arginine side chain is large and positively charged. Citrulline (Cit) is isosteric but neutral and polar, lysine is slightly smaller but also usually positively charged at neutral pH, and glutamine (Q) is smaller and neutral and polar (Fig. 2 *B*). At *A. kurodai* FaNaC FMQFa showed a total reduction in potency (no currents at 100 *μ*M), whereas FMKFa and FM(Cit)Fa showed similar potency to FMRFa, and at *M. fuliginosus* FaNaC1 each of these analogs showed similar potency to FMRFa (Fig. 2 *A*). Together, this shows that M2 hydrophobicity and R3 basicity seem important for FMRFa potency at *A. kurodai* FaNaC, whereas M2 and R3 side-chain identity seems unimportant for FMRFa potency at *M. fuliginosus* FaNaC1.

Finally, substitutions of the C-terminal F4 of FMRFa had substantially different effects on potency at the channels we tested. In *A. kurodai* FaNaC, we found that most F4 substitutions did not lead to substantial changes in potency (Fig. 2). In stark contrast, F4 substitutions abolished, significantly reduced, or had no significant effect on FMRFa potency at *M. fuliginosus* FaNaC1. Although substituting F4 for a smaller hydrophobic valine side chain in FMRVa did not significantly affect peptide potency at *M. fuliginosus* FaNaC1, substituting F4 for a much smaller hydrophobic alanine side chain in FMRAa abolished peptide potency completely (Fig. 2). Similarly, changes to the C terminus of FMRFa, such as Gly-Ser insertion or an O-methyl ester addition, abolished or drastically reduced activation of only *M. fuliginosus* FaNaC1 (Fig. 2).

These experiments reveal substantial differences in the structure-activity relationships between FMRFa and *A. kurodai* FaNaC *cf*. *M. fuliginosus* FaNaC1. FMRFa F1, M2, and to some extent, R3 are important for peptide potency at clade 1 *A. kurodai* FaNaC. In stark contrast, FMRFa F4 strongly determines peptide potency at clade 2 *M. fuliginosus* FaNaC1, where most substitutions can drastically reduce potency.

### Structural comparison of FMRFa binding in *A. californica* FaNaC and *M. fuliginosus* FaNaC1

**Figure 3:**
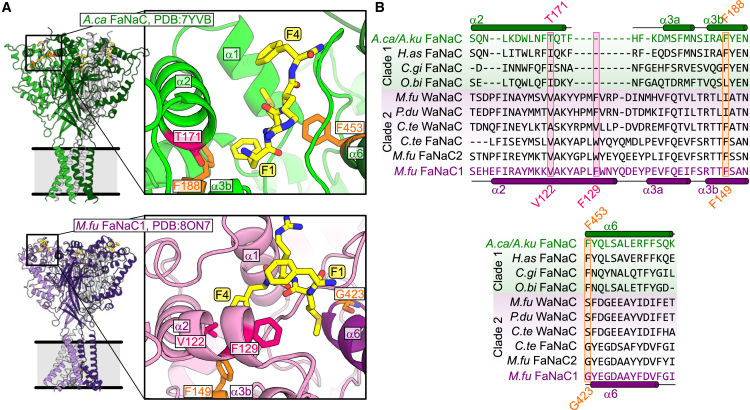
Structural differences between A. *californica* FaNaC and *M. fuliginosus* FaNaC1. (*A*) (*Left*) FMRFa-bound *A. californica* FaNaC (PDB: 7YVB) and *M. fuliginosus* FaNaC1 (PDB: 8ON7) cryo-EM structures; putative lipid bilayer in gray. (*Right*) Magnified view of binding site; side chains in orange and magenta are referred to in (*B*). (*B*) Alignment of several previously characterized FaNaCs from clades 1 and 2. *A.ca*, *Aplysia californica*; *A.ku*, *A. kurodai*; *H.as*, *H. aspersa*; *C.gi*, *Crassostrea gigas*; *O.bi*, *Octopus bimaculoides*; *M.fu*, *M. fuliginosus*; *P.du*, *Platynereis dumerilii*; *C.te*, *Capitella teleta*. Selected amino acid residue numbers shown only for *A. californica* FaNaC and *M. fuliginosus* FaNaC1. *A. californica* FaNaC (shown here) and *A. kurodai* FaNaC (used in our experiments) are 98.5% identical and do not differ in the segments aligned here (Fig. S11).

We compared our experimental analysis with recently published cryo-EM structural analyses of *Aplysia californica* FaNaC (98.5% identical to *A. kurodai* FaNaC used in our experiments; Fig. S11) and *M. fuliginosus* FaNaC1 (14,15). In both structures, the neuropeptide-binding pocket at the external corner of the protein is formed by dynamic *α*1–*α*3 helical segments of the distal “finger domain” and more static *β*6–*β*7 loop and *α*6 helix of proximal-finger and “knuckle” domains (Fig. 3 *A*). However, two striking differences between *A. californica* FaNaC and *M. fuliginosus* FaNaC1 emerge. Firstly, despite similar overall architecture and secondary structure of this site in both channels, *α*1–*α*3 amino acid sequence is remarkably divergent between the two channels, and this pattern extends throughout their clade 1 and clade 2 cousins (Fig. 3 *A* and *B*). Thus, certain FaNaC1 residues that interact with FMRFa and whose mutation decreases FMRFa potency in *M. fuliginosus* FaNaC1 occupy different orientations in space or are absent from *A. californica* FaNaC (e.g., *M. fuliginosus* FaNaC1 F129 in Fig. 3) (15).

Secondly, the peptide orients into the two different channels in different, essentially opposite, binding modes. FMRFa F1 orients most deeply into the site in *A. californica* FaNaC and interacts with *α*3-F188 and *α*6-F453 (Fig. 3 *A*). FMRFa M2 is also buried in the site, close to *α*6-F453. In contrast, FMRFa F4 buries most deeply into the site in *M. fuliginosus* FaNaC1, wedging between *α*3-V122 and *α*3-F129 (Fig. 3 *A*). Thus, our observation that FMRFa activity at *A. kurodai* FaNaC depends on FMRFa F1 and M2 probably derives in part from direct interactions of FMRFa F1 and M2 side chains with FaNaC *α*3-F188 and *α*6-F453 side chains, and our observation that FMRFa activity at *M. fuliginosus* FaNaC1 depends on FMRFa F4 probably derives from direct interactions between the FMRFa F4 side chain and FaNaC1 *α*3-V129 and *α*3-F129 side chains.

### Chemistry of FMRFa interactions with *A. kurodai* FaNaC

We next sought to dissect the nature of these putative interactions by measuring FMRFa potency at *A. kurodai* FaNaC and *M. fuliginosus* FaNaC1 mutants in which FaNaC phenylalanine residues were replaced with noncanonical amino acids (ncAAs) of subtly different physico-chemical properties. To this end, we co-injected oocytes with amber stop codon (UAG) mutant FaNaC mRNAs and UAG-suppressing tRNAs aminoacylated with a desired ncAA (19). Via this “nonsense suppression” approach, we attempted to replace *A. kurodai* FaNaC *α*3-F188 and *α*6-F453, and *M. fuliginosus* FaNaC1 *α*3-F129, with phenylalanine (Phe) itself as a control; cyclohexylalanine (Cha), a nonaromatic analog; and homophenylalanine (hPhe), a similar ring but on a longer aliphatic stem. We also utilized conventional tyrosine substitutions, essentially adding a hydroxyl group to the *para* group of the aromatic ring.

**Figure 4:**
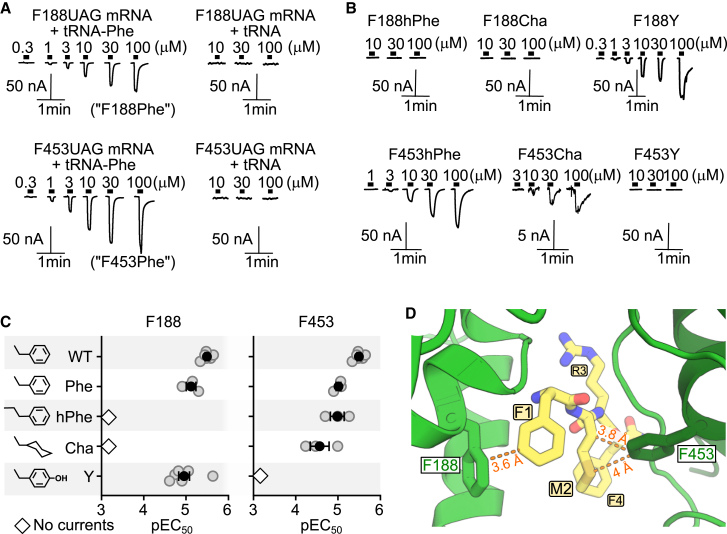
*A. kurodai* FaNaC mutagenesis and nonpolar interactions. (*A*) Nonsense suppression of mutant UAG stop codon in *A. kurodai* FaNaC by acetylated tRNA: example two-electrode voltage clamp recordings of FMRFa-gated currents in oocytes injected with UAG mutant mRNA and either acetylated tRNA (*left panels*) or unacetylated tRNA (*right panels*). (*B*) FMRFa-gated currents in oocytes prepared with indicated nonsense suppression mutants (*hPhe*, homophenylalanine; *Cha*, cyclohexylalanine) or conventional F-Y mutant. (*C*) (*Left*) Phenylalanine (WT and Phe), hPhe, Cha, and tyrosine (Y) side chains. (*Middle and right*) Mean ± SE (*n* = 3–5) pEC_50_ values for FMRFa activation of indicated *A. kurodai* FaNaC mutants. (*D*) Magnified view of FMRFa-binding site in *A. californica* FaNaC (PDB: 7YVB). One subunit light green, one dark green. Selected side chains and intermolecular distances labeled.

Preliminary experiments suggested that ncAAs were effectively incorporated into *A. kurodai* FaNaC *α*3-188 and *α*6-453 positions, as oocytes injected with UAG mutant mRNAs and aminoacylated tRNAs showed FMRFa-gated currents, whereas oocytes injected with UAG mutant mRNAs and un-acylated tRNAs showed no FMRFa-gated currents (Fig. 4 *A*). Curiously, *A. kurodai* FaNaC incorporating phenylalanine itself at *α*3-188 and *α*6-453 positions via nonsense suppression showed decreased FMRFa potency compared to regular WT (F188Phe and F453Phe in Fig. 4 *A* and *C*). This could derive from either re-acylation of tRNAs and nonspecific incorporation of endogenous amino acids after initial Phe incorporation, leading to various mutant proteins, or from altered potency in cases of lesser overall surface expression, as interpreted from smaller maximum current amplitude in the nonsense suppression mutants (compare Fig. 4 *A* and *B* with Fig. 1 *B*). The former seems unlikely, based on the absence of current at oocytes injected with UAG mutant mRNAs and un-acylated tRNAs, and, regarding the latter, we are not aware of peptide potency depending on levels of FaNaC expression. Therefore, we cannot explain the shift in potency in nonsense suppression, and we interpret small shifts in potency cautiously here.

*A. kurodai* FaNaC F188Phe and F188Y channels were activated by FMRFa at micromolar concentrations (Fig. 4 *A–C*). In contrast, F188Cha and F188hPhe channels showed little or no response to FMRFa at concentrations up to 100 *μ*M (Fig. 4 *A*–*C*). Thus, activity was lost via a saturated, nonplanar ring in F188Cha and via an aryl but more distal ring of F188hPhe, and activity was intact with the addition of a polar group to the *para* position on the edge of the side chain. This indicates that a specifically positioned aryl ring is important for the FMRFa-F1/FaNaC-F188 interaction, which is consistent with an F1-to-F188 edge-to-face interaction captured in the cryo-EM structure (orange dashed line in Fig. 4 *D*). The F188hPhe substitution might also obscure this deep part of the binding site via the additional methylene group. This role of F188 is also consistent with a previous conventional mutagenesis study of *A. kurodai* FaNaC, where the F188Y mutation was much better tolerated than the F188V mutation (24).

At the FaNaC *α*6-453 position, the addition of a *para*-hydroxyl group to the aryl side chain via the F453Y mutation abolished FMRFa sensitivity, whereas moving the ring further into the FMRFa-binding site via F453hPhe or converting it to nonplanar cyclohexane via F453Cha had relatively little effect on FMRFa sensitivity (Fig. 4 *A–C*). This is consistent with a van der Waals or hydrophobic interaction between the *β*-carbon of FMRFa M2 and the edge of FaNaC F453 (orange dashed line in Fig. 3 *D*). The FMRFa M2 sulfur atom is also within 4 Å of the FaNaC F453 edge, and this is within the range of previously described edge-on sulfur-aromatic interactions (25).

*M. fuliginosus* FaNaC1 position *α*3-F129 seemed less amenable to nonsense suppression. We observed no FMRFa-gated currents even in oocytes injected with UAG mutant mRNA and phenylalaninylated tRNA (not shown). Furthermore, when we measured oocyte surface expression via a C-terminal c-Myc tag in various FaNaC constructs, we saw little if any surface expression of *M. fuliginosus* FaNaC1 F129 ncAA mutants, in contrast to *A. kurodai* FaNaC 188 and 453 ncAA mutants (Fig. S12). Thus, and despite the fact that conventional F129 A/L/Q mutants were functionally expressed in oocytes, where they showed reduced sensitivity to FMRFa (15), this position or receptor seems resistant to the nonsense suppression method. We have not explored this further.

## Discussion

### Chemical determinants of neuropeptide potency

Our results, together with structural data (14,15), show that the FMRFa F1 side chain is essential for FMRFa potency at the clade 1 FaNaC via its F1-to-F188 edge-to-face interaction. If engineering more potent ligands for such a receptor were desirable, FMRFa analogs in which F1 is replaced with halogenated derivatives could be tested, as certain edge substituents such as this can enhance edge-to-face interactions in certain systems (26). Curiously, the tri-fluorinated F1-containing peptide showed enhanced potency at the clade 2 FaNaC, despite F1 being less essential at FaNaC1, also suggesting a potential route to enhancing potency of ligands at a clade 2 FaNaC via the FMRFa F1 residue.

Like FMRFa F1, M2 is also more important for potency at the clade 1 FaNaC than the clade 2 FaNaC, via interactions with FaNaC F453. This seems perfectly engineered by nature, as it likely involves electrostatic interactions between the electronegative M2 sulfur atom and the partial positive charge of the F453 edge (25) together with van der Waals or hydrophobic interactions between M2 CH_2_ and F453 (Fig. 4 *D*). We were not able to dissect FMRFa M2-FaNaC1 F129 interactions in chemical detail, as our attempts to incorporate ncAAs into *M. fuliginosis* FaNaC1 via nonsense suppression were unsuccessful.

FMRFa F4 and the C-terminal amide are important for potency at clade 2 *M. fuliginosus* FaNaC1 and appear much less important for potency at clade 1 *A. kurodai* FaNaC. In the former, van der Waals or hydrophobic interactions between FMRFa F4 and FaNaC1 F129, together with a short amide tail, may be required for binding. It is notable that FMRVa showed similar potency to FMRFa at FaNaC1, as FVRIamides and also Wamides are capable of activating *M. fuliginosus* FaNaC1 and other clade 2 FaNaCs/WaNaCs, respectively. The fact that valine, isoleucine, phenylalanine, or potentially tryptophan from the peptide agonist can fulfill this role deep in the binding site might suggest that the remainder of the peptide can orient slightly differently into the remainder of the binding site, consistent with the tolerance of clade 2 FaNaC1 for substitutions in F1, M2, and R3 of the peptide.

In contrast, clade 1 *A. kurodai* FaNaC was relatively impervious to FMRFa C-terminal modifications, suggesting that the putative hydrogen bonds between FMRFa C-terminal amide and vicinal main-chain carbonyl oxygen atoms of the receptor in the closely related *A. californica* FaNaC cryo-EM structure (14) are dispensable. However, an earlier report suggested that, e.g., FMRF (no amide) and FRFLa show drastically reduced potency at clade 1 *Helix aspersa* FaNaC compared to FMRFa (27). We cannot explain this discrepancy between *A. kurodai* FaNaC and *H. aspersa* FaNaC, which share 72% amino acid sequence identity (Fig. S11) and an FMRFa EC_50_ of 3–5 *μ*M. However, as 29 out of 31 FMRFa F1, M2, R3, or F4/C-terminal substitutions in the study of *H. aspersa* FaNaC drastically reduced agonist potency (27), perhaps *H. aspersa* FaNaC is uniquely sensitive to perturbations in ligand structure.

### Evolution of neuropeptide receptors

Although there is significant difference in the residues implicated in ligand binding between clade 1 and clade 2 FaNaCs, their overall architecture, the formation of the peptide binding site by *α*1-*α*3, and their gating mechanisms are similar. This is on account of highly conserved residues located toward the core of the protein, between ligand-binding and channel domains (15). Thus, evolution may select for activation by different ligands via mutations in two parts of the channel: the core gating machinery and the peptide binding site. In the case of clade 1 and 2 FaNaCs, it seems that core gating machinery has been strictly retained, whereas constraints on conservation of ligand-binding residues have been released, leading to diverse peptides activating various FaNaCs, particularly in clade 2.

The monophyly of clade 1 and 2 FaNaCs raises the possibility that their last common ancestor was FMRFa gated, but answering this question—and assessing other possibilities, such as FMRFa binding to some channels in both orientations, or an ancestral, as-yet-uncharacterized non-FMRFa ligand activating numerous FaNaCs—would require experimental comparison with a sister branch of channels, branching before the FaNaC branch. At the moment, all we have is a brief study of putative sister channels from phoronids and flatworms that failed to identify an agonist and reported that an FMRFa propeptide is absent in those animals (9). We can at least speculate on the makeup of the ancestral FaNaC peptide-binding site by comparing putative *α*1–*α*3 sequence in clade 1 and 2 FaNaCs with that of channels in the non-FaNaC sister branch. The large alignment in the earlier study shows that the *α*1-*α*3 helices forming the peptide-binding site comprise some 50–60 residues both in clade 1 FaNaCs and in the non-FaNaC, sister-branch of phoronid/flatworm channels (9). In contrast, *α*1-*α*3 helices comprise 70–80 residues in annelid-specific clade 2 channels, perhaps reflected in the longer *α*1-*α*2 loop and *α*2 helix in the clade 2 FaNaC structure (Fig. 3 *A*). This offers tentative evidence that the ancestor of clade 1 and 2 FaNaCs had a peptide binding site more similar to extant clade 1 FaNaCs, whose extant descendants are sensitive to native peptides such as FMRFa, FLRFa, and FMKFa and insensitive to native peptides such as NGHYMRFa, (pyroglutamate)FYRFa, LFRYa, ASSFVRIa, and AWVGDKSLSWa (9,27). In contrast, the *α*1-*α*3 sequence underwent more changes in clade 2 FaNaCs after annelids diverged from mollusks, and extant descendants now bind native peptides such as FMRFa, LFRYa, ASSFVRIa, and AWVGDKSLSWa (9,23,27). It is worth noting that agonists for clade 1 FaNaCs from annelids have not yet been identified (9).

An interesting biological difference between clade 1 and 2 FaNaCs may derive from another biophysical difference between the channels. Although clade 1 FaNaCs are rapidly activated by neuropeptide application and rapidly deactivated after removal of the peptide, channels in clade 2 activate and rapidly desensitize, resulting in long recovery time in some channels. Based on high-resolution *M. fuliginosus* FaNaC1 structures, this involves the collapse of the upper pore and rearrangements of extracellular loops situated between the FMRFa-binding site and the channel pore (15). This could have large consequences for the excitability of cells expressing FaNaCs, as FMRFa-induced excitation may persist in the continued presence of peptide, disappear due to desensitization, or even linger due to slow deactivation depending on FaNaC identity. Whether clade 2 FaNaCs are close to peptide secretory cells, reminiscent of small-molecule transmitters activating and desensitizing vertebrate synaptic receptors, or whether clade 2 FaNaC activity is different due to long diffusion of neuropeptides, would perhaps offer interesting insight into the evolution of synapses (28,29). Reconstructing the co-evolution of FLPs and FaNaCs and similar channels would also be informative regarding the evolution of important signaling systems; however, homology among FLPs is not always clear and their evolution is thus difficult to reconstruct.

## Acknowledgments

This work received funding from The Research Council of Norway (project no. 234817).

## Author contributions

E.J.S.C., M.D., H.Y.H., and T.L. designed the research. E.J.S.C., M.D., and L.L. performed molecular biology, electrophysiological experiments, and data analysis. E.J.S.C. and L.L. performed noncanonical amino acid incorporation. H.Y.H. synthesized noncanonical amino acids. E.J.S.C., H.Y.H., and T.L. prepared figures and wrote the manuscript. All authors read and approved the manuscript.

## Declaration of interests

The authors declare no competing interests.

## Supporting Material

Document S1. Figures S1–S12

Document S2. Article plus supporting material
