# Supplementary material for: Flipped binding modes for the same agonist in closely related neuropeptide-gated ion channels
Source: Biophys J. 2025 Jan 11;124(7):1049–57. doi: 10.1016/j.bpj.2025.01.004 (PMC11993918; doi:10.1016/j.bpj.2025.01.004)
Supplement: Document S1. Figures S1–S12 [file mmc1.pdf]

Biophysical Journal, Volume *124*

## Supplemental information

# Flipped binding modes for the same agonist in closely related neuro-peptide-gated ion channels

**Emily J.S. Claereboudt, Mowgli Dandamudi, Léa Longueville, Hassan Y. Harb, and Timothy Lynagh**

**Supplemental information**

# Flipped binding modes for the same agonist in closely related neuropeptide-gated ion channels

Emily J.S. Claereboudt, Mowgli Dandamudi, Léa Longueville, Hassan Y. Harb, Timothy Lynagh

# Supplemental materials and methods

## Synthesis of aminoacylated dinucleotides

### General

All reactions were carried out using commercial materials and reagents without further purification unless otherwise noted. All reactions were monitored by thin layer chromatography (TLC) on silica gel plates (Keiselgel 60 F254, Merck) and/or ultra-performance liquid chromatography (UPLC). Visualization of the spots on TLC plates was achieved by UV light and by staining the TLC plates in potassium permanganate and charring with a heat gun, unless otherwise stated.

UPLC refers to a Waters Acquity UPLC HClass instrument with Acquity PDA detector, QDA mass detector and quaternary solvent system; PDA: 210-350 nm. Acidic methods were run using varying gradients of acetonitrile and water with 5%  2 vol% formic acid (99%) in water on the following columns: Acquity CSH C18 column (2.1 x 50 mm 1.7 µm) at 0.8 mL/min; Acquity CSH-Phenyl Hexyl (CSH PH) column (2.1 x 50 mm 1.7 µm) ; HSS T3 column (2.1 x 50 mm 1.8 µm) at 0.8 mL/min. Basic methods were run using varying gradients of acetonitrile and water with 5% 2 vol% ammonia (28%) in water on the following columns: XBridge BEH C18 column (2.5 µm 2.1 x 50 mm) at 0.8 mL/min; XBridge BEH C8 column (2.5 µm 2.1 x 50 mm) at 0.8 mL/min. Eluents A: water, B: acetonitrile, C: 2 vol % ammonia (28%) in water, D: 2 vol % formic acid (99%) in water.

Chromatography was performed on a Biotage Isolera using silica (normal phase) (SiliCycle SiliaSep Premium 25 µm or Biotage SNAP Ultra HP-Sphere 25 µm) or C18 (reverse phase) (Biotage SNAP Ultra C18 HP Sphere 25 µm) pre-packed cartridges; or by flash-column chromatography using silica gel (Fluorochem silica gel 60A 40-63 µm).

### General procedure A

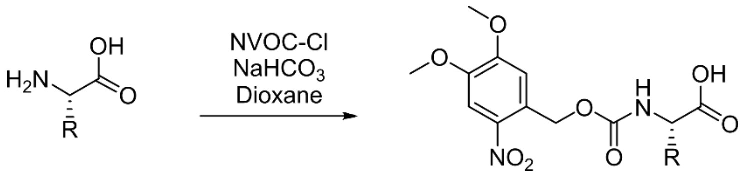


**Figure S1.** A suspension of amino acid (1 eq), (4,5-dimethoxy-2-nitro-phenyl)methyl carbonochloridate (1.1 eq) and sodium hydrogen carbonate (2.5 eq) in 1,4-dioxane (106 eq) was stirred at room temperature overnight. Water was added and stirred at room temp for 30 min. The reaction was diluted with ethyl acetate and the phases separated. The organic phase was discarded. The aqueous was acidified to ~pH 1 with 2 M HCl aqueous solution. The product was extracted with ethyl acetate and dried over $MgSO_4$. The solution was concentrated under vacuum to give the desired product which was used without purification in the next step.

**General procedure B**

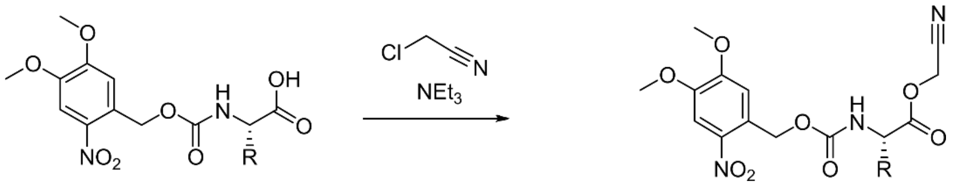


**Figure S2.** A stirring solution of NVOC-amino acid (1 eq) and triethylamine (2 eq) in chloroacetonitrile (50 eq) was stirred at room temp overnight. The reaction mixture was concentrated under vacuum. The residue was suspended in EtOAc and sonicated for 10 min. The mixture was filtered and the filtrate concentrated under vacuum to give the desired cyanomethyl ester. Used in the next step without purification.

**General procedure C**

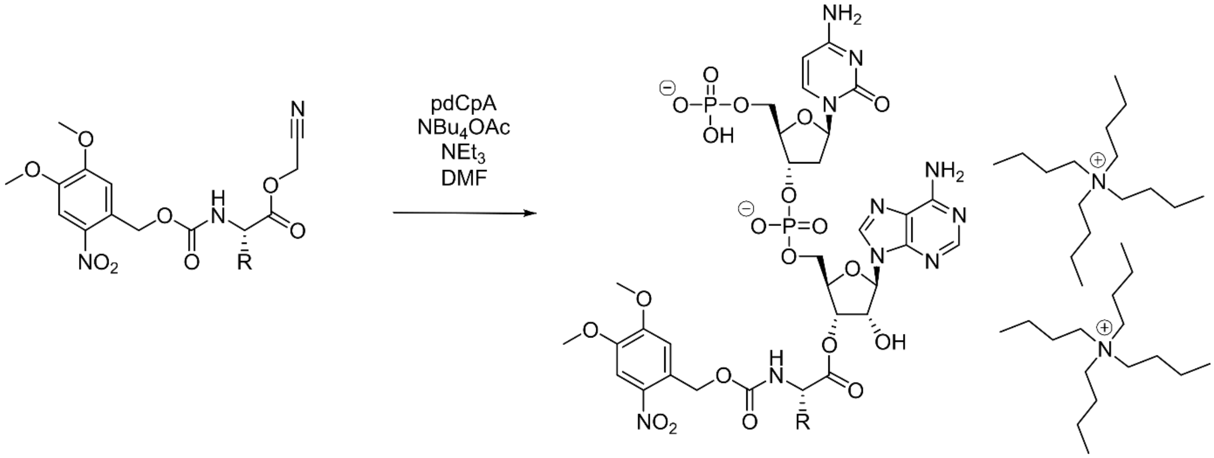


**Figure S3.** A solution of pdCpA (1 eq, synthesized according to (1), cyanomethyl ester (1 eq), tetrabutylammonium acetate (2 eq) and triethylamine (2 eq) in *N*,*N*-dimethylformamide (70 eq) was stirred at 38 °C overnight. The reaction mixture was directly purified by reverse phase purification (Biotage Isolera, 12 g SiliaSep C18 cartridge; gradient 20-70% (acetonitrile + 0.1%formic acid) in (water + 0.1% formic acid) over 12 CV). The product containing fractions were freeze dried overnight to give the desired compound as a mixture of monoacylated diastereomers.

**Ditetrabutylammonium Nvoc-Phe-OpdCpA**

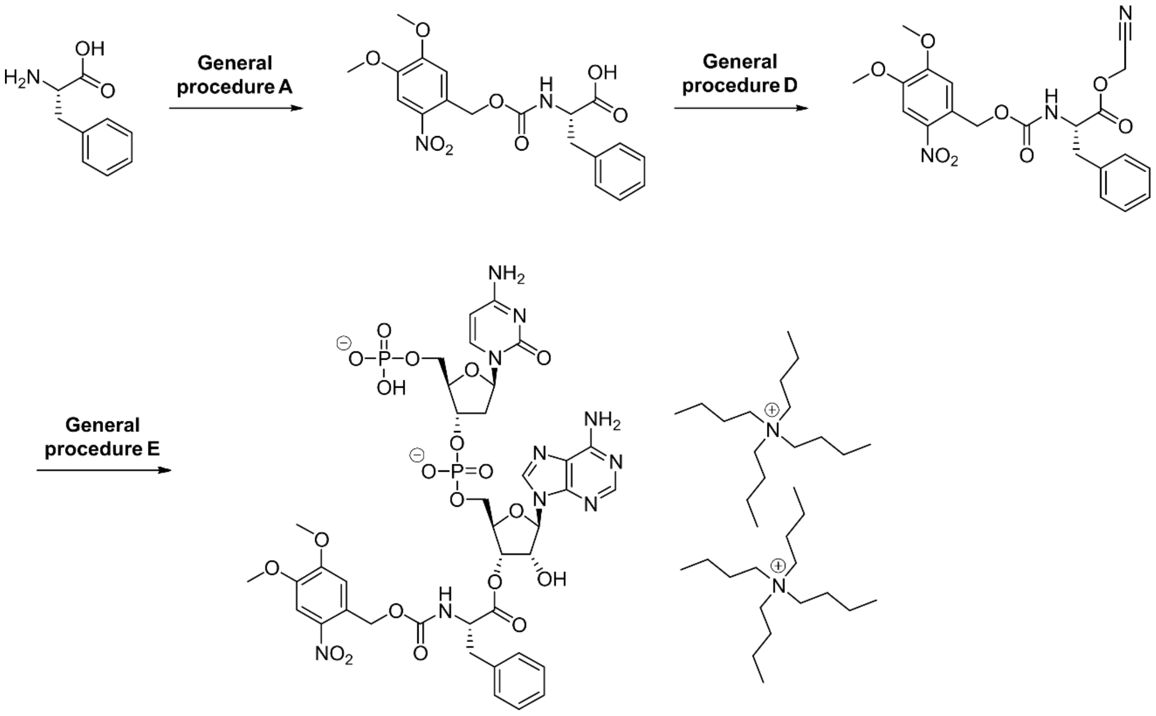


**Figure S4.** Using general procedure A, *L*-phenylalanine (81.7 mg, 0.495) gave the desired product Nvoc-Phe-OH as yellow solid, 199 mg (99%). It was used in the next step without purification. UPLC-MS: (CSH-C18 Short Acid 2 to 95%, 1204-041-1) $R_t$ = 0.88 min (92.2%), MS (ESIpos): m/z = $[M+Na]^+$ 427; MS (ESIneg): m/z = $[M-H]^-$ 403. Using general procedure B, Nvoc-Phe-OH (189 mg, 0.467 mmol) gave the desired product Nvoc-Phe-$OCH_2CN$ as a yellow solid, 184 mg (91%). It was used in the next step without purification. UPLC-MS: (CSH-C18 Short Acid 2 to 95%, 1204-046-1a) $R_t$ = 0.95 min (88.9%), MS (ESIpos): m/z = $[M+Na]^+$ 466. Using general procedure C, Nvoc-Phe-$OCH_2CN$ (12.2 mg, 0.275 mmol) gave the desired product, di-tetrabutylammonium Nvoc-Phe-OpdCpA as a white solid, 7.80 mg (19%). UPLC-MS: (BEH-C18 Long Base 2 to 20%, 1204-053-2b) $R_t$ = 1.42 min (93.4%), MS (ESIpos): m/z = $[M+H]^+$ 1023; MS (ESIneg): m/z = $[M-H]^-$ 1021 (Fig. S7).

**Ditetrabutylammonium Nvoc-hPhe-OpdCpA**

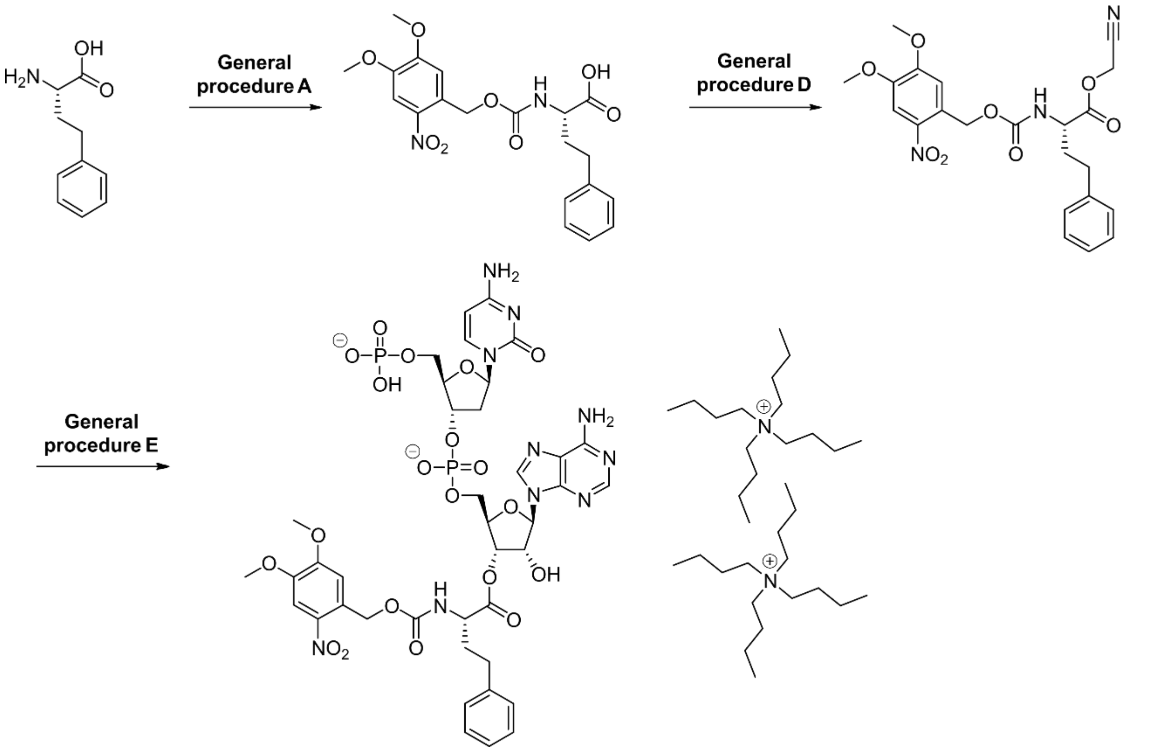


**Figure S5.** Using general procedure A, *L*-homophenylalanine (88.7 mg, 0.495 mmol) gave the desired product Nvoc-hPhe-OH as an orange solid, 78.0 mg (38%). It was used in the next step without purification. UPLC-MS: (CSH-C18 Short Acid 2 to 95%, 1204-069-1) $R_t$ = 0.91 min (88.1%), MS (ESIpos): m/z = $[M+Na]^+$ 441; MS (ESIneg): m/z = $[M-H]^-$ 417. Using general procedure B, Nvoc-hPhe-OH (68.0 mg, 0.163 mmol) gave the desired product Nvoc-hPhe-$OCH_2CN$, 73.0 mg (98%). It was used in the next step without purification. UPLC-MS: (CSH-C18 Short Acid 50 to 95%, 1204-076-1) $R_t$ = 0.49 min (88.5%), MS (ESIpos): m/z = $[M+Na]^+$ 480. Using general procedure C, Nvoc-hPhe-$OCH_2CN$ (21.5 mg, 0.0471 mmol) gave the desired product, di-tetrabutylammonium Nvoc-hPhe-OpdCpA as a white solid, 13.1 mg (18%). UPLC-MS: (BEH-C18 Short Base 2 to 50%, 1104-079-2b) $R_t$ = 0.44 min (95.3%), MS (ESIneg): m/z = $[M-H]^-$ 1035 (Fig. S8).

**Ditetrabutylammonium Nvoc-Cha-OpdCpA**

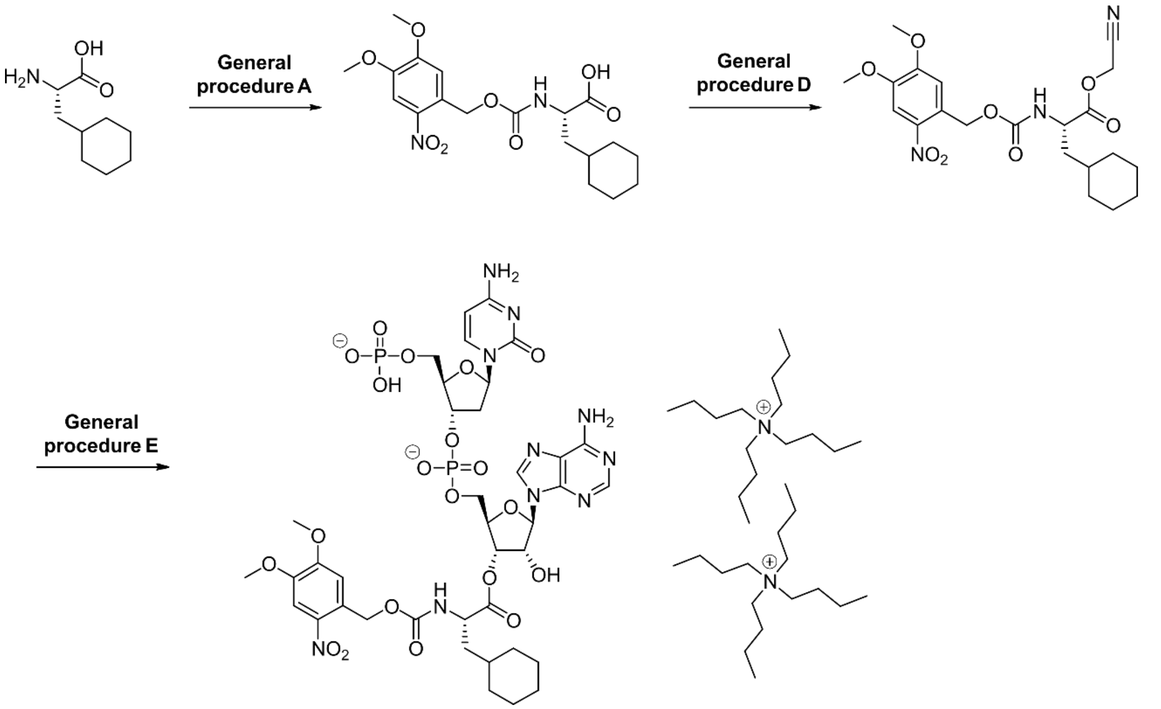


**Figure S6.** Using general procedure A, (S)-2-amino-3-cyclohexylpropanoic acid (84.7 mg, 0.495 mmol) gave the desired product Nvoc-Cha-OH as an orange solid, 102 mg (50%). It was used in the next step without purification. UPLC-MS: (CSH-C18 Short Acid 2 to 95%, 1204-030-1) $R_t$ = 0.89 min (95.0%), MS (ESIpos): m/z = $[M+Na]^+$ 433; MS (ESIneg): m/z = $[M-H]^-$ 409. Using general procedure B, Nvoc-Cha-OH (92.0 mg, 0.224 mmol) gave the desired product Nvoc-Cha-$OCH_2CN$ as a yellow solid, 88.0 mg (87%). It was used in the next step without purification. UPLC-MS: (CSH-C18 Short Acid 50 to 95%, 1204-077) $R_t$ = 0.58 min (96.2%), MS (ESIpos): m/z = $[M+Na]^+$ 472. Using general procedure C, Nvoc-Cha-$OCH_2CN$ (21.2 mg, 0.0471 mmol) gave the desired product, di-tetrabutylammonium Nvoc-Cha-OpdCpA as a white solid, 14.1 mg (20%). UPLC-MS: (BEH-C18 Short Base 2 to 50%, 1104-080-2b) $R_t$ = 0.47 min (97.5%), MS (ESIpos): m/z = $[M+H]^+$ 1029; MS (ESIneg): m/z = $[M-H]^-$ 1027 (Fig. S9).

# Supplemental figures

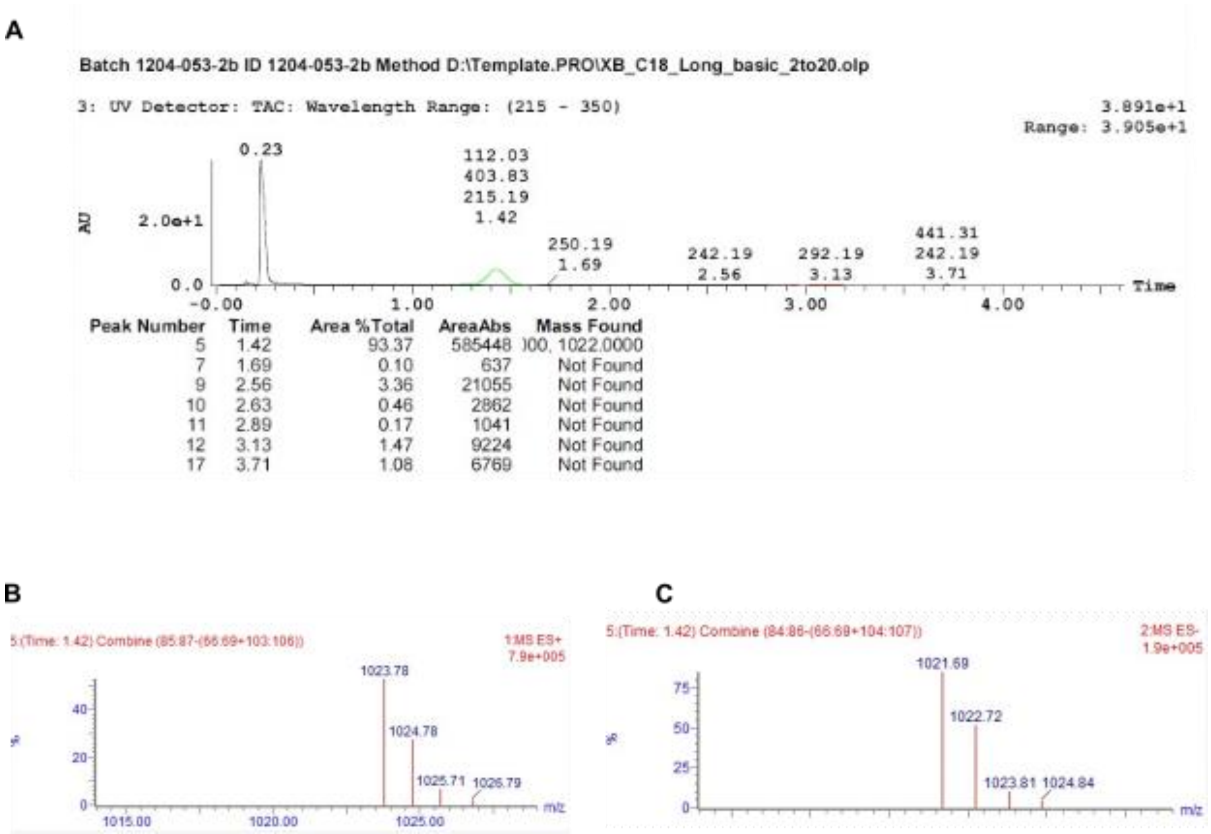


**Figure S7. Characterization of ditetrabutylammonium Nvoc-Phe-OpdCpA**

UPLC-MS results confirming desired product.

**A**

Batch 1104-079-2b ID 1104-079-2b Method C:\MassLynx\XB_C18_Short_basic_2to50.olp

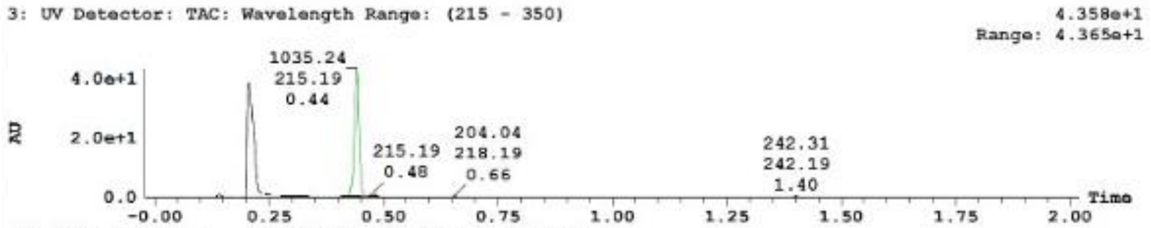


| Peak Number | Time | Area %Total | AreaAbs | Mass Found |
|---|---|---|---|---|
| 1 | 0.44 | 95.28 | 495604 | 1036.0000 |
| 2 | 0.48 | 1.94 | 10069 | Not Found |
| 3 | 0.54 | 0.29 | 1521 | Not Found |
| 4 | 0.66 | 1.27 | 6590 | Not Found |
| 5 | 0.68 | 0.41 | 2120 | Not Found |
| 8 | 1.40 | 0.81 | 4237 | Not Found |

**B**

| Peak ID | Time | BPM |
|---|---|---|
| 1 | 0.43 | 1035.24 |

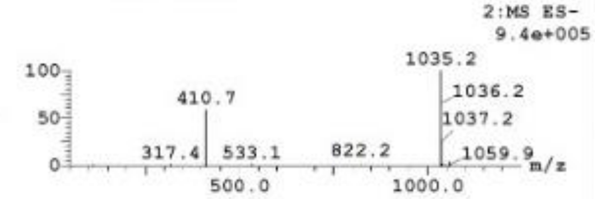


**Figure S8. Characterization of ditetrabutylammonium Nvoc-hPhe-OpdCpA**

UPLC-MS results confirming desired product.

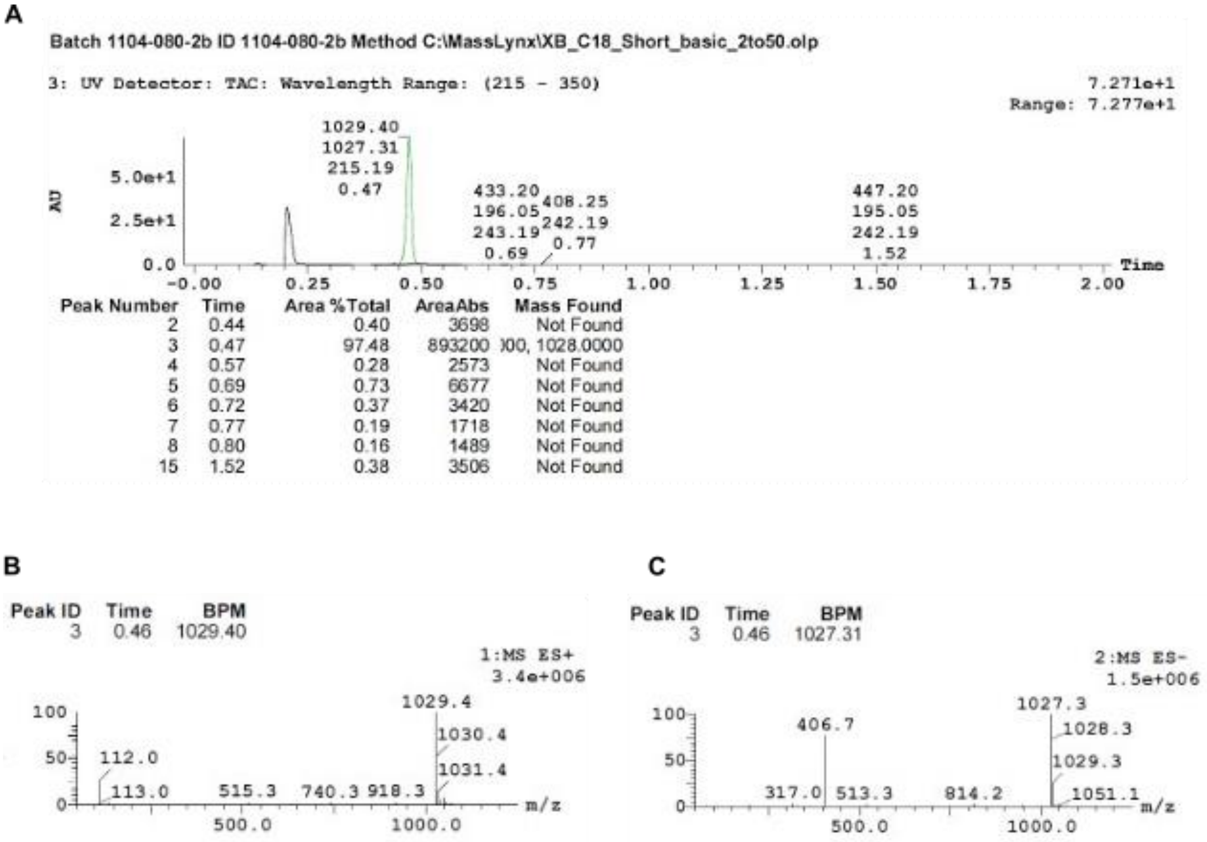


| Peak Number | Time | Area %Total | AreaAbs | Mass Found |
|---|---|---|---|---|
| 2 | 0.44 | 0.40 | 3698 | Not Found |
| 3 | 0.47 | 97.48 | 893200 | [illegible]00, 1028.0000 |
| 4 | 0.57 | 0.28 | 2573 | Not Found |
| 5 | 0.69 | 0.73 | 6677 | Not Found |
| 6 | 0.72 | 0.37 | 3420 | Not Found |
| 7 | 0.77 | 0.19 | 1718 | Not Found |
| 8 | 0.80 | 0.16 | 1489 | Not Found |
| 15 | 1.52 | 0.38 | 3506 | Not Found |

**Figure S9. Characterization of ditetrabutylammonium Nvoc-Cha-OpdCpA**

UPLC-MS results confirming desired product.

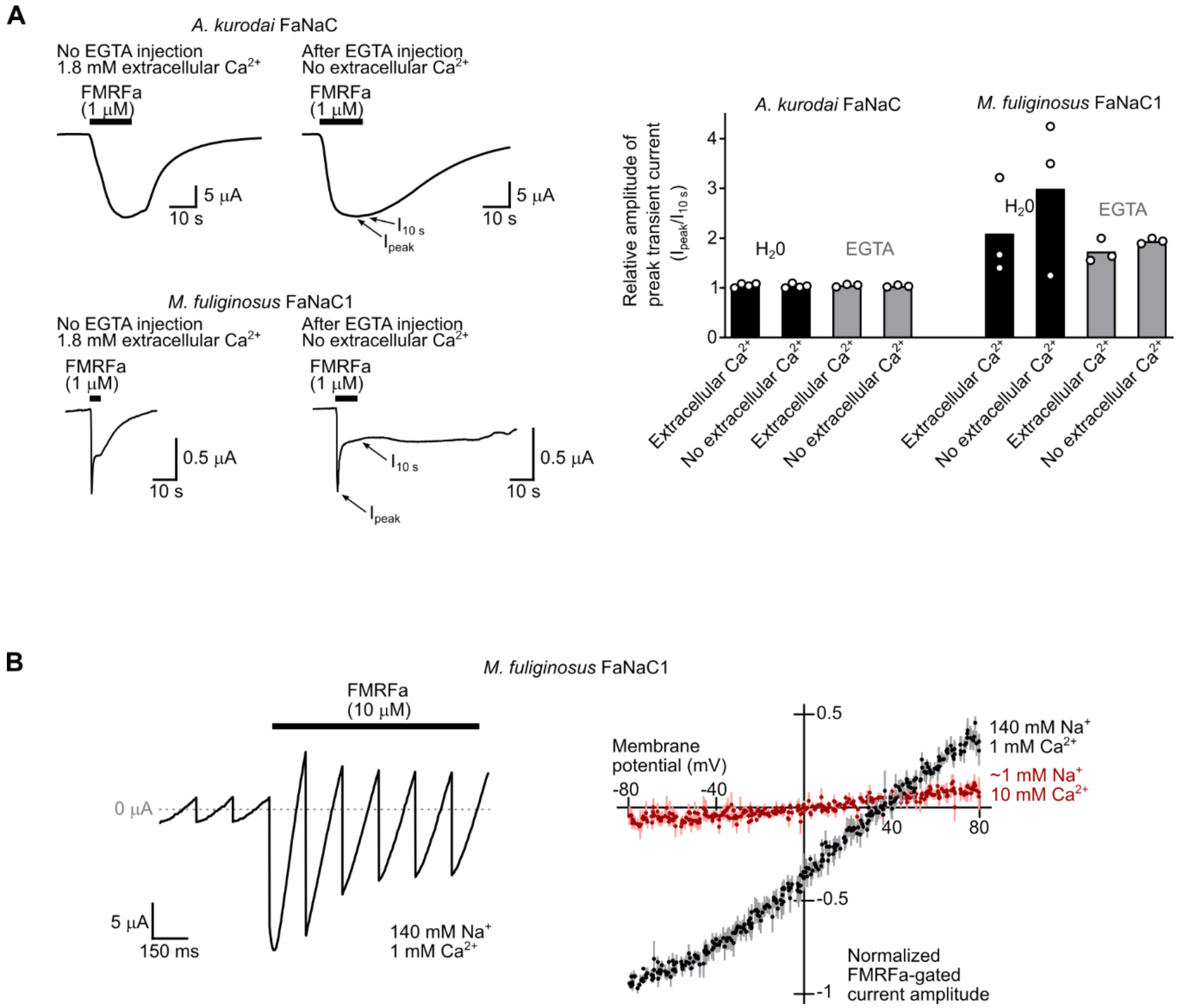


**Figure S10. Tests for potential $Ca^{2+}$ permeability**

(**A**) *Left*, example recordings of oocytes injected with indicated FaNaCs after no EGTA injection and including 1.8 mM $Ca^{2+}$ in the extracellular solution, and after EGTA injection and without extracellular $Ca^{2+}$ in the solution ($Ba^{2+}$ instead), as indicated. *Right*, mean (columns) and data points (dots, n = 3-4) for relative amplitude of peak transient current in $H_2$0-injected and EGTA injected oocytes expressing indicated FaNaCs and with or without $Ca^{2+}$ in the extracellular solution. Peak transient current amplitude was calculated by dividing the peak current amplitude ($I_{peak}$) by the current remaining after 10 s ($I_{10\ s}$). (**B**) *Left*, example FMRFa-gated current in *M. fuliginosus* FaNaC1-expressing oocyte during 150 ms voltage ramps from -80 mV to 80 mV. *Right*, current without FMRFa was subtracted from peak current with FMRFa, and specific FMRFa-gated current was plotted against membrane potential. This was done with different extracellular solutions, as indicated (mean ± SEM, n = 4). $E_{rev,\ 140Na,1mMCa}$ = 38 mV, $E_{rev,{\sim}1Na,10Ca}$ = -2 mV. (~1 mM $Na^{+}$ from adjusting pH with NaOH.)

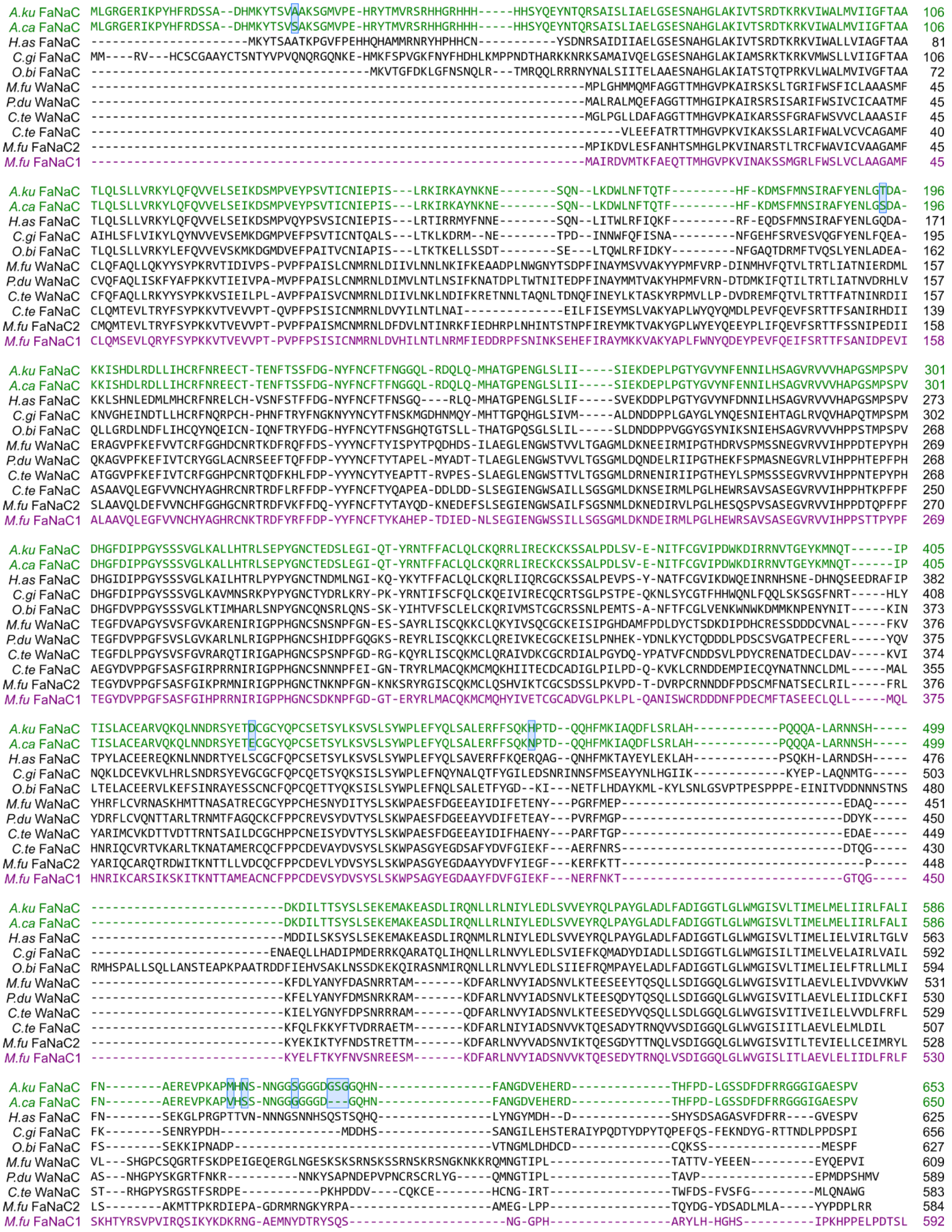


**Figure S11. Full amino acid sequence alignment**

Amino acid sequences of FaNaCs and WaNaCs characterized previously (2-6). Blue highlighting indicates amino acid positions where *Aplysia kurodai* (*A.ku*) FaNaC (used in our experiments) differs from *Aplysia californica* (*A.ca*) FaNaC (used in previous structural studies). *H.as*, *Helix aspersa*; *C.gi*, *Crassostrea gigas*; *O.bi*, *Octopus bimaculoides*; *M.fu*, *Malacoceros fuliginosus*; *P.du*, *Platyneries dumerilii*; *C.te*, *Capitella teleta*.

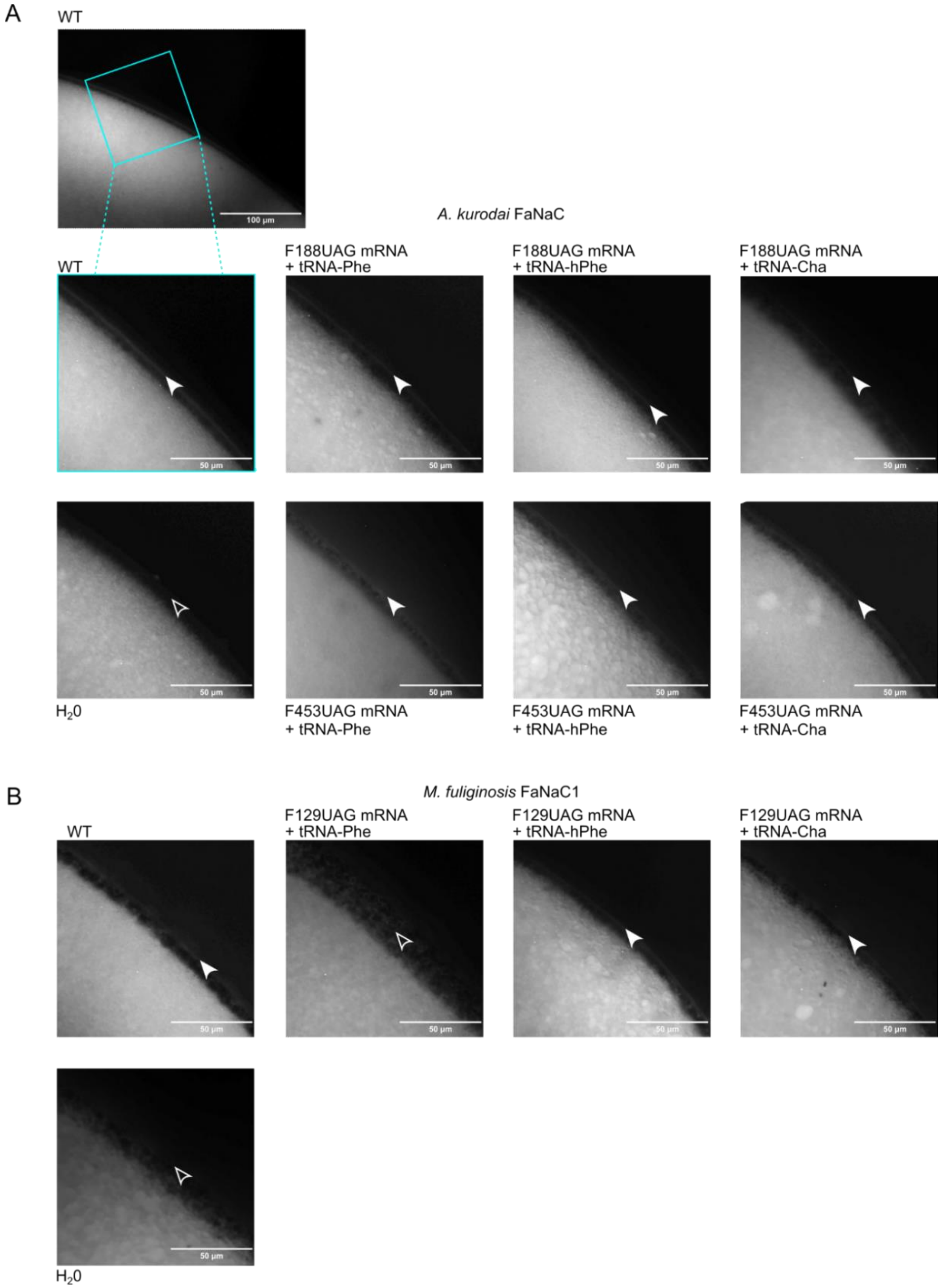


**Figure S12. FaNaC immunolabeling**

(**A**,**B**) Micrographs showing outer surface (arrowheads) of sectioned oocytes after treatment with mouse anti-Myc and fluorescent goat anti-mouse antibodies, after injection with water or *A. kurodai* FaNaC (**A**) or *M. fuliginosus* FaNaC1 (**B**). Scalebars 100 µm (top) or 50 µm (all others). White-filled arrowheads, fluorescent signal on oocyte surface; and black-filled arrowheads, no/weak signal (as interpreted by authors).

## Supplemental references


1. Zhu, X.-F., and A. I. Scott. 2001. An improved synthesis of the dinucleotides pdCpA AND pdCpdA. Nucleosides, Nucleotides & Nucleic Acids 20(3):197-211.
2. Dandamudi, M., H. Hausen, and T. Lynagh. 2022. Comparative analysis defines a broader FMRFamide-gated sodium channel family and determinants of neuropeptide sensitivity. J. Biol. Chem. 298(7):102086.
3. Furukawa, Y., Y. Miyawaki, and G. Abe. 2006. Molecular cloning and functional characterization of the Aplysia FMRFamide-gated Na+ channel. Pflügers Arch. 451(5):646-656.
4. Lingueglia, E., G. Champigny, M. Lazdunski, and P. Barbry. 1995. Cloning of the amiloride-sensitive FMRFamide peptide-gated sodium channel. Nature 378(6558):730-733.
5. Liu, F., Y. Dang, L. Li, H. Feng, J. Li, H. Wang, X. Zhang, Z. Zhang, S. Ye, Y. Tian, and Q. Chen. 2023. Structure and mechanism of a neuropeptide-activated channel in the ENaC/DEG superfamily. Nat. Chem. Biol. 19(10):1276-1285.
6. Schmidt, A., P. Bauknecht, E. A. Williams, K. Augustinowski, S. Grunder, and G. Jekely. 2018. Dual signaling of Wamide myoinhibitory peptides through a peptide-gated channel and a GPCR in Platynereis. Faseb J. 32(10):5338-5349.
